# Case Report: A Unique Case of Pediatric Central Nervous System Embryonal Tumor Harboring the *CIC*–*LEUTX* Fusion, Germline *NBN* Variant and Somatic *TSC2* Mutation: Expanding the Spectrum of *CIC*-Rearranged Neoplasia

**DOI:** 10.3389/fonc.2020.598970

**Published:** 2020-12-02

**Authors:** Wanming Hu, Juan Wang, Li Yuan, Xing Zhang, Yuhang Ji, Chao Song, Jing Zeng, Xiaofei Sun

**Affiliations:** ^1^ Department of Pathology, Sun Yat-sen University Cancer Center, Nanjing, China; ^2^ State Key Laboratory of Oncology in South China, Collaborative Innovation Center of Cancer Medicine, Guangzhou, China; ^3^ Department of Pediatric Oncology, Sun Yat-sen University Cancer Center Medicine, Nanjing, China; ^4^ Department of Pathology, Guangzhou Women and Children Medical Center, Nanjing, China; ^5^ State Key Laboratory of Translational Medicine and Innovative Drug Development, Jiangsu Simcere Diagnostics Co., Ltd, Nanjing, China

**Keywords:** *CIC*, *LEUTX*, next generation sequencing, central nervous system embryonal tumor, *TSC2*, *NBN*

## Abstract

Central nervous system (CNS) embryonal tumors (WHO grade IV) are a heterogeneous group of rare, poorly differentiated neuroepithelial malignant neoplasms that commonly occur in children, and they have a poor prognosis. The 2016 WHO (World Health Organization) classification of CNS tumors created a major shift in paradigm of the classification of embryonal tumors. However, some cases were still difficult to classify. Further integrative genomic analysis is needed to improve the precise classification, diagnosis and treatment of CNS embryonal tumors. Herein, we firstly report a case of CNS embryonal tumor harboring the pathogenic *CIC*–*LEUTX* gene fusion. A 2-year-old male infant presented with a solid cystic mass in the left temporal lobe-basal ganglia and left parietal lobe (maximum diameter, 75 mm) and underwent gross tumor resection. The tumor was classified as a poorly differentiated embryonal neoplasm of neuroectodermal origin that lacked specific features and rosettes. By immunohistochemistry, the tumor cells were strongly positive for synaptophysin, and the Ki67 proliferation index was high (>50%). FISH (Fluorescence in situ hybridization) results indicated no change in the copy number at the 19q13.42 C19MC locus. Next generation sequencing showed a *CIC*–*LEUTX* gene fusion, a somatic *TSC2* c.G2714A mutation, and a heterozygous germline *NBN* c.C127T mutation. One month after surgery, there was recurrence of the intracranial tumor (maximum diameter, 55 mm) as well as spinal cord implantation metastasis. The patient received chemotherapy (CTX+CBP+VCR/DDP+VP-16), radiotherapy, and a drug targeting the *TSC2* gene (everolimus). At the time of this writing, the patient is alive without evidence of disease for 11 months. This is the first report of the *CIC*–*LEUTX* gene fusion in a case of CNS embryonal tumor.

## Introduction

Central nervous system (CNS) embryonal tumors are rare, poorly differentiated neuroepithelial malignant neoplasms that commonly occur in children, including a wide range of aggressive malignancies. The most common clinical manifestations are symptoms and sighs of increased intracranial pressure. However, the overlapping morphological features of these lesions present a diagnostic challenge and undermine the discovery of optimal treatment strategies. A lot of supratentorial CNS embryonal tumors have been histologically classified as primitive neuroectodermal tumor (PNET) in the past, and can have histological features overlapping those of other brain tumors. In recent years, with the advancement of genome technology, the molecular heterogeneity of these tumors is gradually being understood. Some of CNS embryonal tumors have been reclassified from this group through the identification of unique molecular biomarkers, such as embryonal tumors with abundant neuropils and true rosettes, which exhibit alterations in the 19q13.42 C19MC locus, and they are now considered to be a distinct genetically-defined entity known as the embryonal tumor with multilayered rosettes (ETMR), C19MC altered. Another entity has also been identified; embryonal tumors with multilayered rosettes (ETMR), *DICER1*-altered, have mutually exclusive biallelic *DICER1* mutations in which the first hit is typically inherited through the germline (1). However, several CNS embryonal tumors remain unclassified and they are presently defined as CNS embryonal tumors, NOS (not otherwise specific)/NEC (not elsewhere classified). The pathogenesis of these tumors is still unclear. Here, we firstly report the presence of the *CIC*–*LEUTX* gene fusion in a case of CNS embryonal tumor.

### Case Presentation

**Figure 1:**
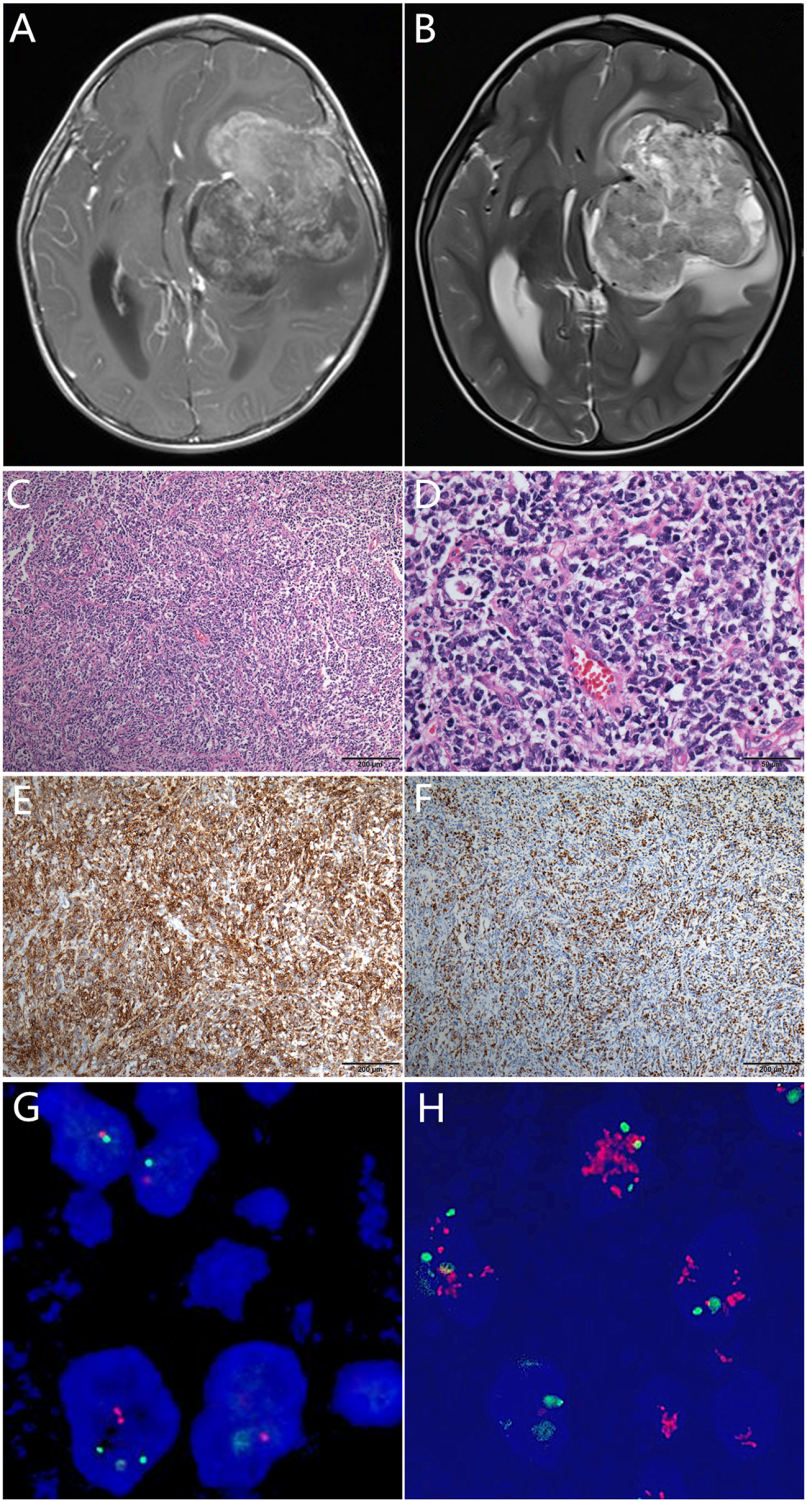
Pre-operative T1-weighted **(A)** and T2-weighted **(B)** magnetic resonance images showing a solid cystic mass with heterogeneous hyperintensity and surrounding edema. Histologically, the tumor was comprised of poorly differentiated/undifferentiated cells with neuropils and proliferated endothelial cells, but no typical true rosettes **(C)**. Some tumor cells were small, round, and poorly-differentiated, whereas some cells presented with atypia as well as rough and dark chromatin. Mitotic figures and apoptotic bodies were easily identified under HPF ×400 **(D)**. Immunohistochemically, the tumor cells were synaptophysin positive **(E)**, and the Ki67 index was >50% **(F)**. The C19MC locus showed no alteration **(G)** compared with the positive amplification positive control **(H)**.

A 2-year-old male infant presented with vomiting and drowsiness lasting for 2 months. On examination, the muscle strength of the right upper limb was grade II and lips skewed. This patient’s pregnancy was of normal length, and his delivery was uncomplicated; his birth weight was 2600g, height 50cm, and his Apgar score is 9. Brain magnetic resonance imaging (MRI) scans revealed a solid cystic mass with lesions in the left temporal lobe-basal ganglia-corona radiate, midline shifted to the right, and nodules in the left parietal lobe (maximum diameter, 75 mm) (**Figures 1A, B**). The patient underwent gross resection of the lesions. Microscopical analysis revealed that the tumor was a poorly differentiated embryonal neoplasm of neuroectodermal origin lacking specific histopathological features. The tumor was comprised of diffuse undifferentiated embryonal cells with neuropils, but no true rosettes (**Figures 1C, D**). Some tumor cells were small, round, and poorly differentiated, whereas other cells presented with atypia as well as rough and dark chromatin. Mitotic figures and apoptotic bodies were easily identified. By immunochemistry, cells were negative for glial fibrillary acidic protein (GFAP) and strongly positive for synaptophysin (Syn) (**Figure 1E**). The Ki67 proliferation index was high (>50%) (**Figure 1F**), and fluorescence in situ hybridization (FISH) results showed no change in the copy number at the 19q13.42 C19MC locus (**Figures 1G, H**). The final diagnosis was CNS embryonal tumor, not elsewhere classified (NEC), according to the guidelines of the revised 2016 World Health Organization Classification of Tumors of the Central Nervous System and cIMPACT-NOW workgroup (2).

**Figure 2:**
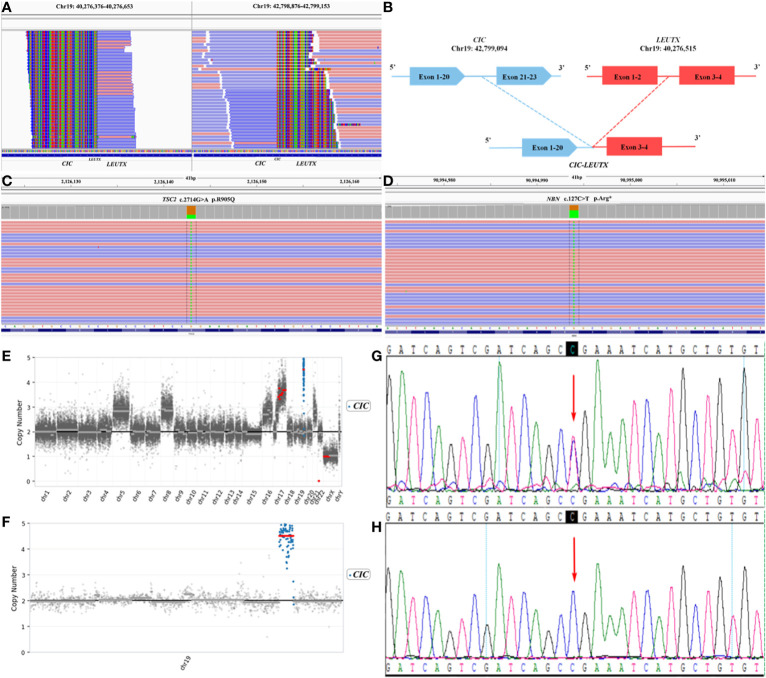
Next generation sequencing (NGS) revealing **(A)** a *CIC*–*LEUTX* gene fusion in exon 20 of the *CIC* gene and exon 3 of the *LEUTX* gene, **(B)** a germline *NBN* c.C127T variant, and **(C)** a somatic *TSC2* c.G2714A variant **(D)**. **(E)** Copy number variation (CNV) of the *CIC* gene was highest in the whole genome. **(F)** The *CIC* gene was amplified 4.85-fold on chromosome 19. **(G)** The unaffected father of the patient carried the heterozygous *NBN* c.C127T mutation, **(H)** whereas the healthy mother did not.

In order to integrate histological and molecular diagnosis, and seek candidate treatments, comprehensive genomic profiling was performed using a 539 cancer related genes panel based on next generation sequencing (NGS) *via* DNA-based hybrid capture in formalin-fixed and paraffin-embedded primary tumor tissue. The panel was sequenced with an average number of mapping reads as 2 megabase and high median depth (> 4000×). We found a fusion of CIC–LEUTX genes with breakpoints located in exon 20 of the CIC gene and exon 3 of the LEUTX gene (**Figures 2A, B**). In addition, a somatic mutation in the TSC2 gene (**Figure 2C**) and a germline mutation in the NBN gene (**Figure 2D**) were also identified. Copy number variation (CNV) showed the CIC gene to be amplified 4.85-fold on chromosome 19 (**Figures 2E, F**). TSC2 encoded tuberin, which was a GTPase-activating protein and regulator of mTORC1 by modulating brain-enriched Rhed (3). The somatic variant c.G2714A located on tuberin-type domain of TSC2, a critical and well-established functional domain, and evaluated as pathogenic mutation by public database ClinVar. The mutation resulted in loss of function of TSC2 leaded to the activation of mTORC1, which regulated the cell growth and survival (4). The patient may benefit from the mTOR inhibitor RAD001 (everolimus), which has been proved an ability to penetrate the blood-brain barrier (5). Further studies by Sanger sequencing revealed that the patient’s father, and not his mother, was a carrier of the NBN heterozygous mutation (**Figures 2G, H**). This patient’s parents and elder sister are healthy. However, his grandfather died of leukemia, and his grandfather diagnosed with bladder cancer.

The patient presented with weakness of the right upper limb five weeks after surgery. Plain and enhanced MRI scans of the brain revealed a partial deletion in the left parietal lobe, the presence of multiple nodules and lesions (maximum diameter, 55 mm) near the surgical site, and left basal ganglia and left basis cranii, indicative of disease recurrence. The left forehead, bilateral temporal region, suprasellar area, front portion of the pons, and pia mater of the bilateral cerebellar hemisphere were unevenly thickened, suggesting implantation metastasis. Plain and enhanced MRI scans of the cervical vertebra revealed lumbo-vertebral canal subdural nodules, slightly thickened terminale meninges, and suspicious lesions that appeared to have spread *via* the cerebrospinal fluid. The patient received chemotherapy (cyclophosphamide+carboplatin +vincristine/cisplatin+etoposide), an mTOR inhibitor (everolimus), and radiotherapy. The tumor shrunk significantly with clinical symptoms relief after therapy. At the time of this writing, the patient was still being treated without any clinical symptoms.

## Discussion and Conclusion

CNS embryonal tumors are aggressive, poorly differentiated brain tumors with poor prognosis and limited effective therapies. Although new subtypes, such as embryonal tumors with multilayered rosettes (ETMR), C19MC altered, and embryonal tumors with multilayered rosettes (ETMR), *DICER1*-altered, have been described, there are few reported cases of new molecular changes in CNS embryonal tumors. In 2016, Dominik Sturm et al. (6) identified several CNS embryonal tumors designated as CNS neuroblastoma with *FOXR2* activation (CNS NB–*FOXR2*), CNS Ewing sarcoma family tumor with *CIC* alteration (CNS EFT–*CIC*), CNS high-grade neuroepithelial tumor with *MN1* alteration (CNS HGNET–*MN1*), and CNS high-grade neuroepithelial tumor with *BCOR* alteration (CNS HGNET–*BCOR*). However, a *CIC*–*NUTM1* gene fusion or a *CIC* frameshift deletion, not a *CIC*–*LEUTX* gene fusion and a *CIC* amplification, were detected in CNS EFT–*CIC*. In review of the literature, we found only three cases of the *CIC*–*LEUTX* gene fusion were reported in CNS, namely a case of CNS angiosarcoma, a case of anaplastic ganglioglioma, and a case of anaplastic astrocytoma with epithelioid GBM features (7, 8) (**Table 1**), and this is the first case of *CIC*–*LEUTX* gene fusion in a CNS embryonal tumor.

**Table 1:** Clinical, histological features and treatment of the tumor with the *CIC*–*LEUTX* gene fusion in central nervous system.

No	Age	Sex	Histologic diagnosis	Molecular changes	Location	Therapy	Follow-up (Month)	Status
1(8)	43	F	angiosarcoma	*CIC*-*LEUTX* and *TSC2*	left tentorial extension to the left midbrain and thalamus	N.A	N.A	N.A
2(7)	12	F	anaplastic astrocytoma with epithelioid GBM features	*CIC*-*LEUTX*	Intraventricular	GTR + RT/TMZ	3	Alive, on therapy
3(7)	19	F	anaplastic ganglioglioma	*CIC*-*LEUTX*	Frontoparietal	GTR	10	Progression
						RT + TMZ → STR + RT (CSI) + carboplatin → cytoxan/ doxo/ifos/etoposide	56	Alive, progressive disease
4	2	M	CNS embryonal tumor	*CIC*-*LEUTX*, *TCS2* and *NBN*	left temporal lobe-basal ganglia and left parietal lobe	PR	1	Progression
						(CTX+CBP+VCR/DDP+VP-16), radiotherapy, and *TSC2* targeted drug (everolimus)	11	Alive

GBM, glioblastoma; CNS, central nervous system; NA, not applicable; GRT, gross total resection; RT, radiotherapy; TMZ, temozolomide; STR, subtotal resection; CSI, craniospinal irradiation; PD, progression disease; doxo, doxorubicin; ifos, ifosfamide; CTX, cyclophosphamide; CBP, carboplatin; VCR, vincristine; DDP, cisplatin; VP-16, etoposide.

We think CNS tumors harboring the *CIC*–*LEUTX* gene fusion may be a new entity. It should be noted that the histological features of these four cases were totally different; however, the gene fusion was identical. Interestingly, both our case and the case of CNS angiosarcoma also harbored the *TCS2* gene mutation apart from *CIC*–*LEUTX* gene fusion, and our targeted therapy was effective. However, it is not known whether the other two cases had similar mutations. The *TSC2* protein participates in the mTOR/AKT pathway, and mutations in its gene commonly arise. This may offer an opportunity for targeted therapies such as everolimus (9). In addition, this is the first report of the *CIC*–*LEUTX* gene fusion in CNS embryonal tumors, which expands the spectrum of *CIC*-rearranged neoplasia. *CIC* rearrangement predominantly occurs in small round cell sarcomas that present as Ewing like sarcomas. For instance, *CIC*–*DUX4* gene fusion sarcomas, a new molecular entity recently described in the 2019 WHO Classification of Tumors of Soft Tissue and Bone, are different from Ewing sarcomas in that more aggressive treatment is needed to improve the overall survival. *CIC*–*DUX4* gene fusion sarcomas account for 22–68% in none *EWSR1*-rearranged small round cell sarcomas (10, 11). In addition to *CIC*–*DUX4* gene fusion Ewing-like sarcomas and *CIC*–*LEUTX* gene fusion in angiosarcomas, other genes, such as *NUTM1*, *NUTM2A*, and *FOXO4*, were also involved in *CIC* gene rearrangement sarcomas (**Table S1**). However, it is not known whether these genes can affect the prognosis of cancer patients harboring *CIC* fusions (12), and further studies are needed.

We speculate that one of the reasons for the *CIC*–*LEUTX* gene fusion in this patient was the fragmentation and instability of the *CIC* chromosome caused by the *NBN* gene mutation. The *NBN* gene is responsible for double-strand DNA damage repair, and heterozygous mutation carriers are at risk for several types of tumors such as CNS relapse of B-cell precursor acute lymphoblastic leukemia (13). Mutations in the *NBN* gene lead to DNA breakage, with abnormal meiosis causing the presence of several CNVs, including the *CIC* gene. Therefore, high-risk populations should be prenatally screened for pathogenic mutations.

Germline variant in *NBN* increases the risk of cancer, however, only one germline mutation in a single allele is not sufficient to induce the tumors (14). The *CIC*–*LEUTX* fusion appears to be the oncogenic alteration. *CIC* gene encodes a transcriptional repressor protein which regulates kinase signaling (10). The hypothesis of the molecular mechanism of oncogenic *CIC*-*NUTM1* fusion in CNS sarcomas is NUTM1 moiety by the recruitment of histone acetyltransferase (HAT) leads to transcriptionally activated of specific *CIC* target gene, similar to *BRD4*-*NUTM1* might block differentiation in midline carcinomas (6). The *LEUTX* gene expresses almost specifically in human embryos and may play a critical role in regulating embryo genome activation (15). *LEUTX* is reported as a fusion partner only with *CIC* and *KAT6A* so far. *LEUTX* fused to *KAT6A* is related to acute myeloid leukemia. Unlike other fusion partners of *KAT6A*, *LEUTX* lacks of the HAT domain but have a homeobox domain like *DUX4* (double homeobox 4), the most common partner of *CIC* fusions (16). This suggests that the *LEUTX* binding partner confers chimeric transcriptional regulatory properties to *CIC* similar to *CIC*-*DUX4*. The transcriptional targets of *CIC*-*DUX4* is well characterized, such as *ETV1*, *ETV4*, and *ETV5*. However, the deregulation of CIC target genes of *CIC*-*LEUTX* remains unclear (17). It is difficult to administer target therapy for oncogenic gene fusions involve transcription factors due to not readily druggable in the direct pharmacologic manner (18). Patients with *CIC*-*DUX4* may respond to IGF-1R inhibitor (19), and epigenetically active drugs are worth to test the efficacy in *CIC*-rearranged neoplasia due to the hypoacetylation and methylation involvement (6, 20). In the further study, the generating and expressing *CIC*-*LEUTX* chimera in primary cells could be used for proving the oncogenic mechanism of the fusion.

In conclusion, we report the first case of a CNS embryonal tumor with *CIC*–*LEUTX* gene fusions which expands the spectrum of *CIC*-rearranged neoplasia, provides the molecular evidence for further classification in CNS embryonal tumors, and provided the practice of personalized, precision medicine. We speculate CNS *CIC*–*LEUTX* gene fusion tumors may represent a new entity. However, it should be further confirmed by a larger cohort.

## Data Availability Statement

The original contributions presented in the study are included in the article/**Supplementary Material**. Further inquiries can be directed to the corresponding authors.

## Ethics Statement

Written informed consent was obtained from the individual(s), and minor(s)’ legal guardian/next of kin, for the publication of any potentially identifiable images or data included in this article.

## Author Contributions

WH, LY, and JZ identified and diagnosed the reported case. XZ, YJ, and CS performed NGS analysis. and WH, JW, JZ, and XS wrote and revised the manuscript with input from all authors. All authors contributed to the article and approved the submitted version.

## Conflict of Interest

XZ, YJ, and CS were employed by Jiangsu Simcere Diagnostics Co., Ltd.

The remaining authors declare that the research was conducted in the absence of any commercial or financial relationships that could be construed as a potential conflict of interest.

## Supplementary Material

Supplementary Table 1 Literature review of sarcomas harboring *CIC* fusions

## Abbreviations

CNS, central nervous system; PNET, primitive neuroectodermal tumor; IHC, immunohistochemistry; FISH, fluorescence in situ hybridization; NGS, next generation sequencing; CNV, copy number variation; NEC, not elsewhere classified; ETMR, embryonal tumor with multilayered rosettes; MRI, magnetic resonance imaging; GFAP, glial fibrillary acidic protein; Syn, synaptophysin; FFPE, formalin-fixed and paraffin-embedded; GBM, glioblastoma; N/A, not applicable; GRT, gross total resection; RT, radiotherapy; TMZ, temozolomide; STR, subtotal resection; CSI, craniospinal irradiation; PD, progression disease; doxo, doxorubicin; ifos, ifosfamide; CTX, cyclophosphamide; CBP, carboplatin; VCR, vincristine; DDP, cisplatin; VP-16, etoposide; HAT, histone acetyltransferase.

## References

[1] Lambo S von Hoff K Korshunov A Pfister SM Kool M ETMR: a tumor entity in its infancy. Acta Neuropathol (2020) 140:249–66. 10.1007/s00401-020-02182-2 PMC7423804 32601913

[2] Louis DN Wesseling P Paulus W Giannini C Batchelor TT Cairncross JG cIMPACT-NOW update 1: Not otherwise specified (NOS) and not elsewhere classified (NEC). Acta Neuropathol (2018) 135:481–4. 10.1007/s00401-018-1808-0 29372318

[3] Ranek MJ Kokkonen-Simon KM Chen A Dunkerly-Eyring BL Vera MP Qeing CU PKG1-modified TSC2 regulates mTORC1 activity to counter adverse cardiac stress. Nature (2019) 566:264–69. 10.1038/s41586-019-0895-y PMC6426636 30700906

[4] Wang T Xie S Luo R Shi L Xing J Two novel TSC2 mutations in renal epithelioid angiomyolipoma sensitive to everolimus. Cancer Biol Ther (2020) 21:4–11. 10.1080/15384047.2019.1665955 31597506 PMC7012169

[5] O’Reilly T McSheehy PM Kawai R Kretz O McMahon L Brueggen J Comparative pharmacokinetics of RAD001 (everolimus) in normal and tumor-bearing rodents. Cancer Chemother Pharmacol (2010) 65:625–39. 10.1007/s00280-009-1068-8 19784839

[6] Sturm D Orr BA Toprak UH Hovestadt V Jones D Capper D New brain tumor entities emerge from molecular classification of CNS-PNETs. Cell (2016) 164:1060–72. 10.1016/j.cell.2016.01.015 PMC5139621 26919435

[7] Lake JA Donson AM Prince E Davies KD Nellan A Green AL Targeted fusion analysis can aid in the classification and treatment of pediatric glioma, ependymoma, and glioneuronal tumors. Pediatr Blood Cancer (2020) 67:e28028. 10.1002/pbc.28028 31595628 PMC7560962

[8] Noch E Nacev B Chan J Wolden S Tap W Antonescu C A 43 year-old woman with primary central nervous system angiosarcoma with CIC-LEUTX gene rearrangement (P3. 6-017). Neurology (2019) 92:P3.6–017.

[9] Parsons DW Roy A Yang Y Wang T Scollon S Bergstrom K Diagnostic yield of clinical tumor and germline whole-exome sequencing for children with solid tumors. JAMA Oncol (2016) 2:616–24. 10.1001/jamaoncol.2015.5699 PMC5471125 26822237

[10] Miettinen M Felisiak-Golabek A Luina CA Glod J Kaplan RN Killian JK New fusion sarcomas: histopathology and clinical significance of selected entities. Hum Pathol (2019) 86:57–65. 10.1016/j.humpath.2018.12.006 30633925 PMC7443750

[11] Sbaraglia M Righi A Gambarotti M Dei TA Ewing sarcoma and Ewing-like tumors. Virchows Arch (2020) 476:109–19. 10.1007/s00428-019-02720-8 31802230

[12] Louis DN Wesseling P Aldape K Brat DJ Capper D Cree IA cIMPACT-NOW update 6: new entity and diagnostic principle recommendations of the cIMPACT-Utrecht meeting on future CNS tumor classification and grading. Brain Pathol (2020) 30:844–56. 10.1111/bpa.12832 PMC8018152 32307792

[13] Tomasik B Pastorczak A Fendler W Bartlomiejczyk M Braun M Mycko M Heterozygous carriers of germline c.657_661del5 founder mutation in *NBN* gene are at risk of central nervous system relapse of B-cell precursor acute lymphoblastic leukemia. Haematologica (2018) 103:e200–3. 10.3324/haematol.2017.181198 PMC5927986 29419426

[14] Robertson JC Jorcyk CL Oxford JT DICER1 Syndrome: *DICER1* Mutations in Rare Cancers. Cancers (2018) 10:143. 10.3390/cancers10050143 PMC5977116 29762508

[15] Jouhilahti EM Madissoon E Vesterlund L Töhönen V Krjutškov K Plaza Reyes A The human PRD-like homeobox gene *LEUTX* has a central role in embryo genome activation. Development (2016) 143:3459–69. 10.1242/dev.134510 PMC5087614 27578796

[16] Sramkova L Cermakova J Kutkova K Zemanova Z Pavlicek P Zuna J Rapidly progressing acute myeloid leukemia with *KAT6A*-*LEUTX* fusion in a newborn. Pediatr Blood Cancer (2020), e28663. 10.1002/pbc.28663 32779858

[17] Yamazaki Y Takazawa Y Antonescu CR Nakamura T *CIC*-*DUX4* Induces Small Round Cell Sarcomas Distinct from Ewing Sarcoma. Cancer Res (2017) 77:2927–37. 10.1158/0008-5472.CAN-16-3351 PMC5488331 28404587

[18] Okimoto RA Wu W Nanjo S Olivas V Lin YK Ponce RK *CIC*-*DUX4* oncoprotein drives sarcoma metastasis and tumorigenesis *via* distinct regulatory programs. J Clin Invest (2019) 129:3401–6. 10.1172/JCI126366 PMC6668665 31329165

[19] Nakai S Yamada S Outani H Nakai T Yasuda N Mae H Establishment of a novel human *CIC*-*DUX4* sarcoma cell line, Kitra-SRS, with autocrine IGF-1R activation and metastatic potential to the lungs. Sci Rep (2019) 9:15812. 10.1038/s41598-019-52143-3 31676869 PMC6825133

[20] Miele E De Vito R Ciolfi A Pedace L Russo I De Pasquale MD DNA Methylation Profiling for Diagnosing Undifferentiated Sarcoma with Capicua Transcriptional Receptor (*CIC*) Alterations. Int J Mol Sci (2020) 21:1818. 10.3390/ijms21051818 PMC7084764 32155762

