# Supplementary material for: Case Report: A Unique Case of Pediatric Central Nervous System Embryonal Tumor Harboring the CIC–LEUTX Fusion, Germline NBN Variant and Somatic TSC2 Mutation: Expanding the Spectrum of CIC-Rearranged Neoplasia
Source: Front Oncol. 2020 Dec 2;10:598970. doi: 10.3389/fonc.2020.598970 (PMC7738346; doi:10.3389/fonc.2020.598970)
Supplement: Supplementary Table 1 — Literature review of sarcomas harboring CIC fusions [file Table_1.docx]

Table S1 Literature review of patients with sarcomas harboring *CIC* fusions

| Type of cancer | *CIC* fusion | Location & References | Reported Cases |
| --- | --- | --- | --- |
| Ewing-like sarcoma | *CIC-DUX4* | brain ([1-3](#_ENREF_1)) | 3 |
|  |  | spinal ([4](#_ENREF_4),[5](#_ENREF_5)) | 2 |
|  |  | soft tissue ([6-33](#_ENREF_6)) | 198 |
|  |  | bone ([3](#_ENREF_3),[8](#_ENREF_8),[13](#_ENREF_13)) | 5 |
|  |  | viscera ([6](#_ENREF_6),[11](#_ENREF_11),[21](#_ENREF_21),[26](#_ENREF_26),[34-36](#_ENREF_34)) | 22 |
|  |  | unavailable ([37-40](#_ENREF_37)) | 51 |
| Ewing-like sarcoma | *CIC-NUTM1* | brain ([41](#_ENREF_41)) | 2 |
|  |  | viscera ([36](#_ENREF_36),[42](#_ENREF_42)) | 2 |
|  |  | soft tissue ([42-44](#_ENREF_42)) | 9 |
| small round cell sarcoma | *CIC-NUTM2A* | neck ([6](#_ENREF_6)) | 1 |
|  |  | buttock ([45](#_ENREF_45)) | 1 |
| small round cell sarcoma | *CIC-FOXO4* | soft tissue ([46-48](#_ENREF_46)) | 3 |
| angiosarcoma | *CIC-LEUTX* | brain ([49](#_ENREF_49),[50](#_ENREF_50)) | 2 |
|  |  | thigh ([51](#_ENREF_51)) | 1 |

Note: The primary lesion locations of soft tissue contained extremities, trunk, trunk wall/body cavities, and head/neck; viscera included kidney/prostate, stomach, intestine, tonsils, and lung, respectively.

**References**

1. Bielle F, Zanello M, Guillemot D, Delgado MG, Bertrand A, Boch AL, Freneaux P, Mokhtari K. Unusual primary cerebral localization of a *CIC*-*DUX4* translocation tumor of the Ewing sarcoma family. Acta Neuropathol. (2014) 128:309-11. 10.1007/s00401-014-1312-0

2. Ito M, Ishikawa M, Kitajima M, Narita J, Hattori S, Endo O, Goto K. A case report of *CIC*-rearranged undifferentiated small round cell sarcoma in the cerebrum. Diagn Cytopathol. (2016) 44:828-32. 10.1002/dc.23520

3. Kao Y, Sung YS, Chen C, Zhang L, Dickson BC, Swanson D, Vaiyapuri S, Latif F, Alholle A, Huang SC, et al.. ETV transcriptional upregulation is more reliable than RNA sequencing algorithms and FISH in diagnosing round cell sarcomas with *CIC* gene rearrangements. Genes Chromosomes Cancer. (2017) 56:501-10. 10.1002/gcc.22454

4. Donahue JE, Yakirevich E, Zhong S, Treaba DO, Lakis NS, Ali SM, La Monte SMD, Mangray S. Primary Spinal Epidural *CIC-DUX4* Undifferentiated Sarcoma in a Child. Pediatr Dev Pathol. (2018) 21:411-7. 10.1177/1093526617707856

5. Yamada S, Muto J, De Leon JCA, Kumai T, Ito K, Murayama K, Hama N, Nakano Y, Satomi K, Arai Y, Shibata T, Inoue T, Nobusawa S, Ichimura K, Hirose Y, Abe M. Primary spinal intramedullary Ewing-like sarcoma harboring *CIC-DUX4* translocation: a similar cytological appearance as its soft tissue counterpart but no lobulation in association with desmoplastic stroma. Brain Tumor Pathol. (2020) 37:111-7. 10.1007/s10014-020-00366-y

6. Mantilla JG, Ricciotti RW, Chen E, Hoch BL, Liu YJ. Detecting disease-defining gene fusions in unclassified round cell sarcomas using anchored multiplex PCR/targeted RNA next-generation sequencing-Molecular and clinicopathological characterization of 16 cases. Genes Chromosomes Cancer. (2019) 58:713-22. 10.1002/gcc.22763

7. Tardio JC, Machado I, Navarro L, Idrovo F, Sanzortega J, Pellin A, Llombartbosch A. Ewing-like sarcoma with *CIC-DUX4* gene fusion in a patient with neurofibromatosis type 1. A hitherto unreported association. Pathology Research and Practice. (2015) 211:877-82. 10.1016/j.prp.2015.08.003

8. Antonescu CR, Owosho AA, Zhang L, Chen S, Deniz K, Huryn JM, Kao Y, Huang SC, Singer S, Tap WD, et al.. Sarcomas with *CIC*-rearrangements are a distinct pathologic entity with aggressive outcome: a clinicopathologic and molecular study of 115 Cases. The American Journal of Surgical Pathology. (2017) 41:941-9. 10.1097/PAS.0000000000000846

9. Tang S, Dodd LG. *CIC*-*DUX4* sarcoma diagnosed by fine-needle aspiration cytology: A case report. Diagn Cytopathol. (2018) 46:958-63. 10.1002/dc.24027

10. Loke BN, Lee V, Sudhanshi J, Wong MK, Kuick CH, Puhaindran ME, Chang KTE. Novel exon–exon breakpoint in *CIC-DUX4* fusion sarcoma identified by anchored multiplex PCR (Archer FusionPlex Sarcoma Panel). J Clin Pathol. (2017) 70:697-701. 10.1136/jclinpath-2016-204247

11. Specht K, Sung Y, Zhang L, Richter GHS, Fletcher CDM, Antonescu CR. Distinct transcriptional signature and immunoprofile of *CIC‐DUX4* fusion–positive round cell tumors compared to *EWSR1*‐rearranged ewing sarcomas: Further evidence toward distinct pathologic entities. Genes Chromosomes Cancer. (2014) 53:622-33. 10.1002/gcc.22172

12. Krskova L, Stejskalova E, Kabickova E, Mrhalova M, Kodet R. A t(4;19) pediatric undifferentiated sarcoma with a novel variant of the *CIC-DUX4* fusion transcript. Pathol Res Pract. (2017) 213:281-5. 10.1016/j.prp.2016.12.005

13. Gambarotti M, Benini S, Gamberi G, Cocchi S, Palmerini E, Sbaraglia M, Donati D, Picci P, Vanel D, Ferrari S, et al.. *CIC*–*DUX4* fusion-positive round-cell sarcomas of soft tissue and bone: a single-institution morphological and molecular analysis of seven cases. Histopathology. (2016) 69:624-34. 10.1111/his.12985

14. Haidar A, Arekapudi SR, Demattia F, Abuisa E, Kraut M. High-grade undifferentiated small round cell sarcoma with t(4;19)(q35;q13.1) *CIC-DUX4* fusion: emerging entities of soft tissue tumors with unique histopathologic features – a case report and literature review. American Journal of Case Reports. (2015) 16:87-94. 10.12659/AJCR.892551

15. Tsukamoto Y, Futani H, Yoshiya S, Watanabe T, Kihara T, Matsuo S, Hirota S. Primary undifferentiated small round cell sarcoma of the deep abdominal wall with a novel variant of t(10;19) *CIC-DUX4* gene fusion. Pathol Res Pract. (2017) 213:1315-21. 10.1016/j.prp.2017.06.008

16. La Vega LLD, Hovelson DH, Cani AK, Liu CJ, Mchugh JB, Lucas DR, Thomas DG, Patel RM, Tomlins SA. Targeted next-generation sequencing of *CIC-DUX4* soft tissue sarcomas demonstrates low mutational burden and recurrent chromosome 1p loss. Hum Pathol. (2016) 58:161-70. 10.1016/j.humpath.2016.09.004

17. Italiano A, Sung YS, Zhang L, Singer S, Maki RG, Coindre J, Antonescu CR. High prevalence of *CIC* fusion with double-homeobox (*DUX4*) transcription factors in *EWSR1*-negative undifferentiated small blue round cell sarcomas. Genes Chromosomes Cancer. (2012) 51:207-18. 10.1002/gcc.20945

18. Panagopoulos I, Gorunova L, Bjerkehagen B, Heim S. The “Grep” command but not FusionMap, FusionFinder or ChimeraScan captures the *CIC-DUX4* fusion gene from whole transcriptome sequencing data on a small round cell tumor with t(4;19)(q35;q13). PLoS One. (2014) 9:e99439. 10.1371/journal.pone.0099439

19. Chebib I, Jo VY. Round cell sarcoma with *CIC*‐*DUX4* gene fusion: Discussion of the distinctive cytomorphologic, immunohistochemical, and molecular features in the differential diagnosis of round cell tumors. Cancer Cytopathol. (2016) 124:350-61. 10.1002/cncy.21685

20. Ko JS, Marusic Z, Azzato EM, Farkas DH, Van Arnam JS, Seiwerth S, Fritchie KJ, Patel RM, Rubin BP, Billings SD. Superficial Sarcomas with *CIC* Rearrangement Are Aggressive Neoplasms: A Series of Eight Cases. J Cutan Pathol. (2020) 47:509-16. 10.1111/cup.13656

21. Mangray S, Somers GR, He J, Zhong S, Shago M, Treaba DO, Lombardo KA, Ali SM, Yakirevich E. Primary undifferentiated sarcoma of the kidney harboring a novel variant of *CIC-DUX4* gene fusion. Am J Surg Pathol. (2016) 40:1298-301. 10.1097/PAS.0000000000000688

22. Tamada H, Kobayashi M, Sano K, Uehara T, Matsumoto Y, Tateishi A, Ohya M, Kito M, Aoki K, Kanno H. Ultrastructure of *CIC-DUX4* sarcoma: the first pathological report. Ultrastruct Pathol. (2020) 44:1-8. 10.1080/01913123.2020.1737610

23. Lehane F, Tsikleas G, Bettington A, Limarporn K, Wilkinson L, Lehane K. "Cyst" on the forearm of a 28‐year‐old female: Case report of a *CIC*‐rearranged sarcoma. J Cutan Pathol. (2019) 46:599-602. 10.1111/cup.13478

24. Smith SC, Buehler D, Choi E, Mchugh JB, Rubin BP, Billings SD, Balzer B, Thomas DG, Lucas DR, Goldblum JR., et al.. *CIC*-*DUX* sarcomas demonstrate frequent *MYC* amplification and ETS-family transcription factor expression. Mod Pathol. (2015) 28:57-68. 10.1038/modpathol.2014.83

25. Kajtar B, Tornoczky T, Kalman E, Kuzsner J, Hogendoorn PCW, Szuhai K. CD99-positive undifferentiated round cell sarcoma diagnosed on fine needle aspiration cytology, later found to harbour a *CIC*-*DUX4* translocation: a recently described entity. Cytopathology. (2014) 25:129-32. 10.1111/cyt.12079

26. Owosho AA, Estilo CL, Huryn JM, Zhang L, Fletcher CDM, Antonescu CR. Head and Neck Round Cell Sarcomas: A Comparative Clinicopathologic Analysis of 2 Molecular Subsets: Ewing and *CIC*-Rearranged Sarcomas. Head Neck Pathol. (2017) 11:450-9. 10.1007/s12105-017-0808-z

27. Karanian-Philippe M, Velasco V, Longy M, Floquet A, Arnould L, Coindre J, Naouresmear CL, Averous G, Guyon F, Macgrogan G, et al.. *SMARCA4* (*BRG1*) loss of expression is a useful marker for the diagnosis of ovarian small cell carcinoma of the hypercalcemic type (ovarian rhabdoid tumor): A comprehensive analysis of 116 rare gynecologic tumors, 9 soft tissue tumors, and 9 melanomas. Am J Surg Pathol. (2015) 39:1197-205. 10.1097/PAS.0000000000000475

28. Ricker CA, Berlow NE, Crawford KA, Georgopapadakos T, Huelskamp AN, Woods AD, Dhimolea E, Ramkissoon SH, Spunt SL, Rudzinski ER, Keller C. Undifferentiated small round cell sarcoma in a young male: a case report. Cold Spring Harb Mol Case Stud. (2020) 6:a004812. 10.1101/mcs.a00481

29. Richkind KE, Romansky SG, Finklestein JZ. T(4;19) (Q35;Q13.1): A recurrent change in primitive mesenchymal tumors? Cancer Genet Cytogenet. (1995) 87:71-4. 10.1016/0165-4608(95)00240-5

30. Somers GR, Shago M, Zielenska M, Chan HSL, Ngan BY. Primary subcutaneous primitive neuroectodermal tumor with aggressive behavior and an unusual karyotype: case report. Pediatr Dev Pathol. (2004) 7:538-45. 10.1007/s10024-004-2024-6

31. Yoshimoto M, Graham C, Chiltonmacneill S, Lee E, Shago M, Squire JA, Zielenska M, Somers GR. Detailed cytogenetic and array analysis of pediatric primitive sarcomas reveals a recurrent *CIC–DUX4* fusion gene event. Cancer Genet Cytogenet. (2009) 195:1-11. 10.1016/j.cancergencyto.2009.06.015

32. Graham C, Chiltonmacneill S, Zielenska M, Somers GR. The *CIC-DUX4* fusion transcript is present in a subgroup of pediatric primitive round cell sarcomas. Hum Pathol. (2012) 43:180-9. 10.1016/j.humpath.2011.04.023

33. Choi E, Thomas DG, Mchugh JB, Patel RM, Roulston D, Schuetze SM, Chugh R, Biermann JS, Lucas DR. Undifferentiated small round cell sarcoma with t(4;19)(q35;q13.1) *CIC*-*DUX4* fusion a novel highly aggressive soft tissue tumor with distinctive histopathology. Am J Surg Pathol. (2013) 37:1379-86. 10.1097/PAS.0b013e318297a57d

34. Camille A, Annesophie B, Cecile P, Severine B, Gaelle P, Olivier D, Geraldine S, Leerflorin AM, Eleni N, Christian P, et al.. Sarcoma with *CIC*-*DUX4* gene fusion: case report of kidney tumor location in a 12-year-old boy. Pediatr Dev Pathol. (2018) 21:406-10. 10.1177/1093526617706818

35. Bergerat S, Barthelemy P, Mouracade P, Lang H, Saussine C, Lindner V, Jacqmin D. Primary *CIC*-*DUX4* round cell sarcoma of the kidney: A treatment-refractory tumor with poor outcome. Pathol Res Pract. (2017) 213:154-60. 10.1016/j.prp.2016.11.015

36. Mangray S, Kelly DR, Leguellec S, Fridman E, Aggarwal S, Shago M, Matoso A, Madison R, Pramanik S, Zhong S, et al.. Clinicopathologic features of a series of primary renal *CIC*-rearranged sarcomas with comprehensive molecular analysis. Am J Surg Pathol. (2018) 42:1360-9. 10.1097/PAS.0000000000001098

37. Charville GW, Wang WL, Ingram DR, Roy A, Thomas DG, Patel RM, Hornick JL, De Rijn MV, Lazar AJ. *EWSR1* fusion proteins mediate *PAX7* expression in Ewing sarcoma. Mod Pathol. (2017) 30:1312-20. 10.1038/modpathol.2017.49

38. Machado I, Navarro L, Pellin A, Navarro S, Agaimy A, Tardio JC, Karseladze AI, Petrov SV, Scotlandi K, Picci P, et al.. Defining Ewing and Ewing-like small round cell tumors (SRCT): The need for molecular techniques in their categorization and differential diagnosis. A study of 200 cases. Ann Diagn Patho. (2016)l 22:25-32. 10.1016/j.anndiagpath.2016.03.002

39. Baldauf MC, Orth MF, Dallmayer M, Marchetto A, Gerke JS, Rubio RA, Kiran MM, Musa J, Knott MML, Ohmura S, et al.. Robust diagnosis of Ewing sarcoma by immunohistochemical detection of super-enhancer-driven *EWSR1*-*ETS* targets. Oncotarget. (2018) 9:1587-601. 10.18632/oncotarget.20098

40. Hung YP, Fletcher CDM, Hornick JL. Evaluation of NKX2-2 expression in round cell sarcomas and other tumors with *EWSR1* rearrangement: imperfect specificity for Ewing sarcoma. Mod Pathol. (2016) 29:370-80. 10.1038/modpathol.2016.31

41. Sturm D, Orr BA, Toprak UH, Hovestadt V, Jones D, Capper D, Sill M, Buchhalter I, Northcott PA, Leis I, Marcel K, et al.. New Brain Tumor Entities Emerge from Molecular Classification of CNS-PNETs. Cell. (2016) 164:1060-72. 10.1016/j.cell.2016.01.015

42. Loarer FL, Pissaloux D, Watson S, Godfraind C, Galmicherolland L, Silva K, Mayeur L, Italiano A, Michot A, Pierron G, et al.. Clinicopathologic Features of *CIC-NUTM1* Sarcomas, a New Molecular Variant of the Family of *CIC*-Fused Sarcomas. Am J Surg Pathol. (2019) 43:268-76. 10.1097/PAS.0000000000001187

43. Schaefer I, Dal Cin P, Landry LM, Fletcher CDM, Hanna GJ, French CA. *CIC*‐*NUTM1* fusion: A case which expands the spectrum of *NUT*‐rearranged epithelioid malignancies. Genes Chromosomes Cancer. (2018) 57:446-51. 10.1002/gcc.3

44. Watson S, Perrin V, Guillemot D, Reynaud S, Coindre J, Karanian M, Guinebretiere J, Freneaux P, Loarer FL, Bouvet M, et al.. Transcriptomic definition of molecular subgroups of small round cell sarcomas. J Pathol. (2018) 245:29-40. 10.1002/path.5053

45. Sugita S, Arai Y, Aoyama T, Asanuma H, Mukai W, Hama N, Emori M, Shibata T, Hasegawa T. *NUTM2A*-*CIC* fusion small round cell sarcoma: a genetically distinct variant of CIC-rearranged sarcoma. Hum Pathol. (2017) 65:225-30. 10.1016/j.humpath.2017.01.012

46. Sugita S, Arai Y, Tonooka A, Hama N, Totoki Y, Fujii T, Aoyama T, Asanuma H, Tsukahara T, Kaya M, et al.. A novel *CIC*-*FOXO4* gene fusion in undifferentiated small round cell sarcoma: a genetically distinct variant of Ewing-like sarcoma. Am J Surg Pathol. (2014) 38:1571-6. 10.1097/PAS.0000000000000286

47. Solomon DA, Brohl AS, Khan J, Miettinen M. Clinicopathologic features of a second patient with Ewing-like sarcoma harboring *CIC-FOXO4* gene fusion. Am J Surg Pathol. (2014) 38:1724-5. 10.1097/PAS.0000000000000335

48. Brohl AS, Solomon DA, Chang WW, Wang J, Song YK, Sindiri S, Patidar R, Hurd L, Chen L, Shern JF (2014) The genomic landscape of the Ewing sarcoma family of tumors reveals recurrent *STAG2* mutation. PLoS Genetics 10:e1004475. 10.1371/journal.pgen.1004475

49. Huang S-C, Zhang L, Sung Y-S, Chen C-L, Kao Y-C, Agaram NP, Singer S, Tap WD, D’Angelo S, Antonescu CR. Recurrent *CIC* gene abnormalities in angiosarcomas: a molecular study of 120 cases with concurrent investigation of *PLCG1*, *KDR*, *MYC*, and *FLT4* gene alterations. Am J Surg Pathol. (2016) 40:645. 10.1097/PAS.0000000000000582

50. Noch E, Nacev B, Chan J, Wolden S, Tap W, Antonescu C, Khakoo Y. A 43 year-old woman with primary central nervous system angiosarcoma with *CIC-LEUTX* gene rearrangement (P3. 6-017). Neurology. (2019) 92:P3. 6-017.

51. Lake JA, Donson AM, Prince E, Davies KD, Nellan A, Green AL, Mulcahy Levy J, Dorris K, Vibhakar R, Hankinson TC, et al.. Targeted fusion analysis can aid in the classification and treatment of pediatric glioma, ependymoma, and glioneuronal tumors. Pediatr Blood Cancer. (2019) 67:e28028. 10.1002/pbc.28028
